# Progress of regulatory RNA in small extracellular vesicles in colorectal cancer

**DOI:** 10.3389/fcell.2023.1225965

**Published:** 2023-07-12

**Authors:** Xinyi Hu, Yukang Lu, Jiajun Zhou, Lanfeng Wang, Mengting Zhang, Yiping Mao, Zhiping Chen

**Affiliations:** ^1^ The First School of Clinical Medicine, Gannan Medical University, Ganzhou, China; ^2^ Department of Laboratory Medicine, First Affiliated Hospital of Gannan Medical University, Ganzhou, China; ^3^ Department of Nephrology, First Affiliated Hospital of Gannan Medical University, Ganzhou, China

**Keywords:** extracellular vesicles, renal cell carcinoma, tumor microenvironment, engineered extracellular vesicles, tumor vaccine

## Abstract

Colorectal cancer (CRC) is the second most common malignant tumor of the gastrointestinal tract with the second highest mortality rate and the third highest incidence rate. Early diagnosis and treatment are important measures to reduce CRC mortality. Small extracellular vesicles (sEVs) have emerged as key mediators that facilitate communication between tumor cells and various other cells, playing a significant role in the growth, invasion, and metastasis of cancer cells. Regulatory RNAs have been identified as potential biomarkers for early diagnosis and prognosis of CRC, serving as crucial factors in promoting CRC cell proliferation, invasion and metastasis, angiogenesis, drug resistance, and immune cell differentiation. This review provides a comprehensive summary of the vital role of sEVs as biomarkers in CRC diagnosis and their potential application in CRC treatment, highlighting their importance as a promising avenue for further research and clinical translation.

## 1 Introduction

Colorectal cancer (CRC) is a prevalent malignancy affecting the gastrointestinal tract, with mortality and incidence rates ranking second and third among all cancers, respectively. It accounts for roughly 10% of all estimated cases and deaths of CRC in men and women worldwide (Bray et al., 2018). Arises from precursor lesions such as adenomatous polyps or sessile serrated lesions (Shaukat A and TR. 2022), and its incidence is influenced by genetic, environmental, and lifestyle factors such as obesity, smoking, and alcohol consumption (Zhang HW. et al., 2021). Additionally, CRC is associated with the male gender and advanced age (Dekker et al., 2019). Currently, available early screening methods for CRC include colonoscopy, fecal immunohistochemistry test (FIT), fecal occult blood test (FOBT), carcinoembryonic antigen (CEA), carbohydrate antigen 19–9 (CA19-9) (Bakhsh et al., 2022), and septin9 gene methylation blood test (Xiao et al., 2020). Among these, colonoscopy is widely used as the gold standard for early screening of CRC due to its high specificity and sensitivity (Bakhsh et al., 2022). However, it is invasive and may cause bleeding and perforation (Shaukat A and TR. 2022). As CRC typically presents no obvious early symptoms, it is often diagnosed in late stages (Li X. et al., 2022), resulting in a high mortality rate in advanced stages (Xiao et al., 2020). Therefore, early detection, diagnosis, and treatment of CRC are crucial to improving patient outcomes and quality of life.

Small extracellular vesicles (sEVs) are membrane structures surrounded by a lipid bilayer (Shao et al., 2020). These vesicles are secreted by all types of cells in nanoscale biological vesicles with a diameter ranging from 30–100 nm (Mao et al., 2021). They are widely present in various body fluids such as blood, amniotic fluid, urine, malignant ascites, cerebrospinal fluid, breast milk, saliva, lymph, and bile (Umwali et al., 2021). sEVs carry various substances such as RNA, DNA, proteins, sugars, and lipids (Pegtel and Gould, 2019). Despite their small size, sEVs contain a range of cellular components including RNA, DNA, proteins, and lipids; specific proteins within sEVs can provide diagnostic and prognostic information, and the lipids determine the unique rigidity and resistance to deformation of sEVs (Mashouri et al., 2019; Zhang H. et al., 2020; Xie et al., 2022). The composition of sEVs varies depending on the cell type (Zhu et al., 2020), and these components are transported and exchanged between cells via sEVs (Zhang and Yu, 2019), playing a key role in facilitating intercellular communication and tumor progression (Akoto and Sharanjiot. 2021).

Non-coding RNAs (ncRNAs) are functional RNA molecules that can be classified into two major categories: basic structural RNAs (Housekeeping ncRNAs) and regulatory RNAs (RncRNAs). In recent years, RncRNAs, especially miRNA, lncRNA, and circRNA, have been widely studied in small extracellular vesicles (sEVs). This review aims to present the latest research progress of RncRNAs in sEVs (sEV-RncRNAs) in CRC and discuss their potential applications in the diagnosis, development, and treatment of CRC. The goal is to facilitate the early clinical application of related technologies and improve the quality of life for CRC patients.

## 2 Small extracellular vesicles

**FIGURE 1:**
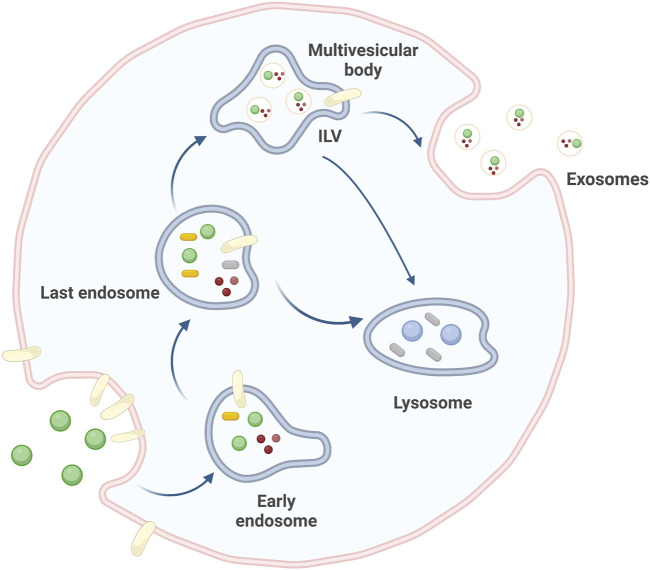
Source pathways of sEVs. sEVs originate from cellular invagination, where EEs continue to mature toward LEs to form ILVs, which eventually drain into the extracellular matrix to form sEVs. Created by BioRender.com.

Extracellular vesicles (EVs), which were first discovered in 1983, are a family of membrane-encapsulated vesicles secreted by cells into the extracellular environment (Wu et al., 2017). This family of vesicles is divided into three subtypes, including small extracellular vesicles (sEVs), microvesicles (MVs), and apoptotic bodies (ABs) (Doyle and MZ, 2019). sEVs are essential mediators of intercellular communication in various physiological and pathological processes. They originate from multivesicular bodies (MVBs), which contain transferrin receptors, and extracellular components such as proteins, lipids, metabolites, small molecules, ions, and fluid. Through endocytosis of the plasma membrane, this mixture of molecules is transported from early endosomes (EEs) to late endosomes (LEs), where the endosomes continue to mature. The LEs are partially invaginated and budded into the surrounding lumen with cell mass content to form intraluminal vesicles (ILVs). These ILVs can be transported and eventually degraded by lysosomes or fused with lipid membranes and released into the extracellular matrix to form sEVs (Théry et al., 2002; Rashed et al., 2017; Gonda et al., 2019, Michel, 2019) (Figure 1). sEVs biogenesis and secretion are complex processes that involve the formation of endosomal-sorting complexes required for transport (ESCRT). The ESCRT machinery consists of four subcomplexes: ESCRT-0, ESCRT-I, ESCRT-II, and ESCRT-III. These subcomplexes work together to sort and package specific cargo proteins into intraluminal vesicles (ILVs) within multivesicular bodies (MVBs), which are eventually released as sEVs. ESCRT-0 plays a crucial role in the sorting of ubiquitinated cargo proteins into lipid structural domains within the endosomal membrane. ESCRT-I and ESCRT-II are then recruited to induce membrane deformation, which forms stable membrane necks that allow for the budding of ILVs into the MVB lumen. Finally, ESCRT-III, along with the Vps4 complex, is recruited to drive vesicle neck breakage and dissociation, which leads to the release of ILVs as sEVs and the recirculation of the ESCRT-III complex (Yue et al., 2020).

Initially, sEVs were thought to be mere cellular waste disposal units and were believed to only replace lysosomes to explain mature erythrocytes. However, further studies of sEVs in the mid-1990s revealed that they had other critical functions (Nabariya et al., 2020). The role of sEVs may vary depending on different physiological or pathological conditions (Li et al., 2018), and their biological origin (Banizs et al., 2014), For example, sEVs obtained from healthy cells may be utilized as drug delivery carriers or immunomodulators (Kim et al., 2021), while sEVs of tumor cell origin may act as carriers of pro-oncogenes that are secreted into recipient cells (Li and Li. 2022), thereby inducing a range of human diseases such as atherosclerosis (Heo and Kang, 2022) and even cancer (Mohammad et al., 2022). The signals from sEVs can be transmitted to the recipient cell by mechanisms such as endocytosis, direct membrane fusion, and receptor-ligand interactions, with endocytosis being the primary method. The exosomal membrane can fuse with the plasma membrane to deliver contents to the recipient cell or bind to homologous receptors on the recipient cell membrane, subsequently triggering an intracellular signaling cascade (Yue et al., 2020).

sEVs are of particular interest due to their small size, which enables them to cross the blood-brain barrier more easily than larger vesicles. This property makes sEVs an attractive vehicle for targeted drug delivery to specific cells or tissues (Wu et al., 2017; Zhao H. et al., 2020). Indeed, sEVs have been utilized as cargo carriers for delivering cancer drugs for targeted therapy (Arrighetti et al., 2019; Gonda et al., 2019) (Table 1). In addition to their potential therapeutic applications, sEVs play a critical role in tumor growth and metastasis by forming “tumor ecological niches” in target organs (Zhao et al., 2018). Cancer-derived sEVs carry biological information that stimulates cancer cell growth and participates in the carcinogenic process (Yu et al., 2021). Therefore, sEVs can also be used as a novel cancer marker (Kok and Yu, 2020). The unique properties of sEVs, including their ability to cross biological barriers and transport specific cargo molecules, make them a promising tool for various biomedical applications. Further research is needed to better understand the mechanisms underlying the biogenesis and function of sEVs, as well as their potential diagnostic and therapeutic applications in the field of cancer research.

**TABLE 1:** sEVs are a useful drug delivery system.

Pharmaceutical cargo	Targeting ligand	Target cells	Role	Reference
5-Fluorouracil (5-FU) and miR-21 inhibitor oligonucleotide (miR-21i)	zHER affibody	CRC HCT-116	Upregulation of chemoresistance to improve treatment efficiency in CRC	Liang et al. (2020b)
SOX2 siRNA	Tlyp-1	non-small cell lung cancer, A549 stem cells	Gene delivery for cancer therapy	Bai et al. (2020)
Imatinib	IL-3	chronic myelogenous leukemia cells	Inhibit cancer cell growth	Bellavia et al. (2017)
BCR-ABL siRNA				
miR-1915-3p	PFKFB3	HT29 and HCT116 cell lines	Improves the chemotherapeutic efficacy of oxaliplatin in CRC	Xiao et al. (2021)
	USP2			
miRNA-26a	ApoA-1	Hepatocellular carcinoma (HepG2)	Suppress tumor cell migration and proliferation	Liang et al. (2018)

## 3 sEV-RncRNAs

ncRNA is a functional RNA molecules that have received extensive attention in the field of sEVs (Li T. et al., 2021). Among ncRNAs, miRNAs are the smallest, endogenous single-stranded ncRNAs of 18–25 nt length (Du et al., 2018; Toden et al., 2021). miRNAs are present in animal and plant genes (Isaac et al., 2021) and are involved in mediating post-transcriptional gene expression (Yu et al., 2016). miRNA is highly conserved and easily preserved, making it one of the most abundant RNAs among sEVs. The biological activity of miRNA is extremely stable and easily detected due to the protection of the envelope, which makes it a biomarker for disease diagnosis and prediction (Yue et al., 2020; Wang D. et al., 2022). In addition to their diagnostic potential, miRNAs can also act as negative regulatory genes that bind to target mRNAs (Li et al., 2022b), promoting cell proliferation, invasion, and metastasis (Hsiuying, 2020).

lncRNAs are a class of ncRNAs that are greater than 200 nucleotides in length and lack a complete open reading frame. They have limited protein-coding ability but are capable of participating in a variety of cellular processes, including chromatin organization, gene transcription, post-transcriptional regulation, and protein translation (Yue et al., 2020; Liu D. et al., 2021). Although lncRNAs are not as easy to preserve as miRNAs, they exhibit strong tissue specificity and can serve as markers for disease prediction and diagnosis (Wang J. et al., 2022). Additionally, lncRNAs function as transcriptional sponges (Yue et al., 2020), regulating gene expression through a variety of mechanisms. They can act as miRNA decoys to prevent target mRNA degradation (Yan and Bu, 2021). In sEVs, lncRNAs are carried and upon entering target cells, regulate target gene expression through sponge miRNAs, which in turn regulate core molecules and signaling pathways that mediate colorectal carcinogenesis and progression (Sun H. et al., 2021).

CircRNA is a type of RNA that possesses a closed-loop structure without a 5′cap and 3′polytail. It can act as both a miRNA sponge and interact with RNA-binding protein (RBP) to encode proteins and regulate transcriptional processes (Zhang X. et al., 2020). Initially, circRNA was thought to be formed by RNA misplacing of exon transcripts. However, as research progressed, circRNA was found to be abundant and structurally stable compared to miRNA and lncRNA (Long et al., 2021). Its special closed structure makes it resistant to nucleic acid exonuclease processing (Wang L. et al., 2022), and it has a longer half-life and higher stability than other RNAs (Li A. et al., 2021). CircRNA is widely expressed in a variety of human cells and can regulate the expression of mRNAs through competitive binding with miRNAs (Yang K. et al., 2022). It can also serve as a biomarker for metastatic CRC (Wang X. et al., 2020).

## 4 Progress of sEV-RncRNAs in the diagnosis of CRC

CRC to human health should not be underestimated. Due to the difficulty in detecting CRC in the early stages and the high mortality rate in the later stages, early detection and treatment are of utmost importance (Zygulska and Pierzchalski, 2022). Conventional assays for CRC have low sensitivity and are invasive, highlighting the need for a highly sensitive, minimally invasive assay with easy access to test specimens. In recent years, research on small extracellular vesicles (sEVs) in CRC has intensified, and an increasing number of studies have found that sEVs can be one of the most promising detection objects in liquid biopsy. This approach can be applied to various aspects of CRC, such as metastasis, drug resistance, and immunomodulation, providing information for early diagnosis of CRC (Zhu et al., 2020) (Table 1). sEVs are present in various cells, with cancer cells containing higher levels of sEVs than normal cells (Lei et al., 2022). sEVs are rich in biomarkers for disease diagnosis and prognosis, and sEVs carry biologically active substances that are differentially expressed in healthy individuals and tumor patients. The detection of biomarkers predictive of cancer in tumor sEVs through liquid biopsy can help improve the specificity and sensitivity of early tumor diagnosis (Zhang et al., 2020d). In the case of CRC screening, sEV-RncRNAs have higher sensitivity and specificity than other blood markers (Baassiri et al., 2020), improving the detection rate of tumor mutations in blood samples (Yu et al., 2021) (Table 2). The abundant content and structural characteristics of miRNAs, lncRNAs, and circRNAs in sEVs make them potential biomarkers for CRC and play an essential role in the early diagnosis of CRC (Table 3).

**TABLE 2:** Advantages of sEv-RncRNas instead of blood markers.

sEv-RncRNAs	Advantages	Cargoes	Role	References
miRNA	High true-positive rate	miR-23 (95.5%) miR-88a (92.0%)	Diagnostic	Ogata-Kawata et al. (2014)
	Low false-positive rate	miR-150 (8.0%) miR-1229 (6.0%)	Diagnostic	Ogata-Kawata et al. (2014)
lncRNA	Highly tissue-specific	UCA1(100%)	Diagnostic	Barbagallo et al. (2018)
circRNA	High sensitivity	circ-0004771 (80.91%)	Diagnostic	Pan et al. (2019)
	High specificity	circ-0004771 (82.86%)	Diagnostic	Pan et al. (2019)

**TABLE 3:** sEV-RncRNAs as biomarkers of CRC and preclinical evidence.

EVs	Source	Cargoes	Number of CRC patients	Expression	Type of biomarker	Role	References
sEV-miRNA	Tissue	miR-224-5p	20	Upregulated	Diagnostic	Promotes value-added invasion of CRC cells	Wu et al. (2022)
		miR-26a	21	Downregulated	Diagnostic	Promoting malignant behavior of CRC cells	Xu et al. (2021b)
		miR-26b					
	Plasma	miR-125a-3p	50	Upregulated	Diagnostic	Improving the ability to diagnose CRC early	Wang et al. (2017)
		miR-92b	40	Upregulated	Diagnostic	Improved diagnostic accuracy of CRC	Min et al. 2019)
		miR-16-5p	13	Downregulated	Diagnostic	As a biomarker of CRC occurrence	Ostenfeld et al. (2016)
		miR-23b-3p					
		miR-27b-3p					
	Serum	miR-1539	51	Upregulated	Diagnostic	Predicting Adverse Clinicopathological Behavior in Tumors	Cui et al. (2020)
		miR-377-3p	173	Downregulated	Diagnostic	Good diagnostic efficiency as a circulating biomarker for CRC diagnosis	Wang et al. (2022a)
sEV-lncRNA	Tissue	lncRNA NNT-AS1	40	Upregulated	Diagnostic	Inhibition of CRC cell proliferation	Yin et al. (2021)
		lncRNA ZEB2-AS1	60	Upregulated	Diagnostic	Induction of apoptosis in CRC cells	Wu et al. 2020)
	Serum	lncRNA UCA1	10	Upregulated	Diagnostic	Promoting cetuximab resistance in CRC with therapeutic implications	Yuan et al. (2022)
		lncRNA HOTTIP	49	Upregulated	Diagnostic	Enhancement of mitomycin resistance	Chen et al. (2020a)
sEV-circRNA	Tissue	circ PABPC1	3	Upregulated	Diagnostic	Promoting CRC progress	Li et al. (2022b)
		circRNA_0001178 circRNA_0000826	40	Upregulated	Diagnostic	Potential to diagnose liver metastases from colorectal cancer	Xu et al. (2019)
		circ_006229		Downregulated	Diagnostic	Induced colorectal cancer follow-up progress	Jin et al. (2018)
	Serum	circ-0004771	170	Upregulated	Diagnostic	Can be used as a diagnostic biomarker for CRC	Pan et al. (2019)

## 5 Role of sEV-RncRNAs in CRC progression

### 5.1 Promote proliferation, invasion, and metastasis of CRC cells

**FIGURE 2:**
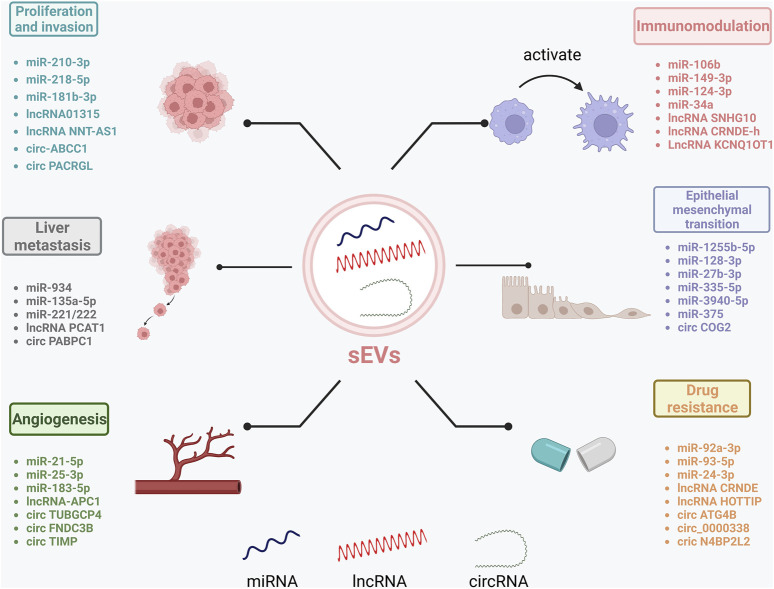
Role of sEV-RncRNAs in CRC progression. sEV-RncRNAs play a role in CRC by promoting proliferation and invasion, promoting liver metastasis, promoting angiogenesis, promoting epithelial-mesenchymal transition, participating in immune regulation, and inducing the development of drug and radioresistance. Created by BioRender.com.

Research shows that ncRNAs play important roles in CRC pathology. sEVs deliver ncRNAs to recipient cells and can regulate CRC proliferation, invasion, and metastasis due to their specificity and sensitivity (Sun H. et al., 2021) (Figure 2).

Malignant progression and poor prognosis of tumors are closely associated with hypoxia in the tumor microenvironment (TME). Ge et al. (2021) discovered that hypoxic colorectal cancer (CRC) cell-derived miR-210-3p can be transferred to normoxic CRC cells through sEVs, inducing pro-tumorigenic effects in the normoxic CRC cells by downregulating CELF2 expression and remodeling the TME of CRC. CELF2 is a tumor suppressor gene, and overexpression of CELF2 can attenuate the promotion of cell proliferation by miR-210-3p and inhibit CRC cell proliferation. Moreover, the hypoxic microenvironment promotes high enrichment of miR-410-3p in sEVs and their delivery to normoxic cells, which enhances CRC tumor progression by targeting PTEN and activating the PI3K/Akt pathway (Hu et al., 2020). Jiang et al. (2019) discovered that miR-181b-3p derived from tumor-associated fibroblasts (CAFs) could promote the pathological process of CRC by regulating the expression of sorting nexin 2 (SNX2) in CRC cells. Furthermore, Liu et al. (2020) found that sEV-mi-106b-3p upregulates the expression of Deleted in liver cancer-1 (DLC-1) in the sera of metastatic CRC patients and promotes the metastasis of CRC. In a recent study by Lan et al. (2019), miR-21-5p and miR-155-5p were found to be upregulated in M2 macrophage-derived sEVs (MDE). Conversely, BRG2, a key factor in promoting colorectal cancer (CRC) metastasis, was downregulated in metastatic CRC cells. These findings suggest that miR-21-5p and miR-155-5p are transferred to CRC cells through MDE, suppressing BRG1 expression and promoting CRC cell metastasis. Furthermore, recent studies further support the critical role of sEVs in CRC progression and metastasis. Zhang X. et al. (2020) demonstrated that the distribution of CAFs was associated with enhanced CRC metastasis. Specifically, miR-17-5p was found to be highly expressed in CAFs-derived sEVs and promoted CRC invasion and metastasis by activating the TGF-β signaling pathway and thus activating CAFs. In addition, Xu F. et al. (2020) found that metastatic CRC-derived sEVs could promote CRC metastasis and tumorigenesis by downregulating miR-26a and miR-26b expression and increasing Fucosyltransferase 4 and fucosylation expression in native cells and activating the PI3K/AKT/mTOR pathway. Specifically, sEVs carrying MALAT1 were able to transfer to native CRC cells and promote metastasis and tumorigenesis. A recent experimental study by Wang D. et al. (2022) provides further evidence for the critical role of sEVs in CRC progression and metastasis. Specifically, it was found that miR-146a-5p and miR-155-5p, derived from sEVs in CRC cells, could promote the activation of CAFs through the JAK2-STAT2/NF-κB signaling pathway and ultimately induce CRC lung metastasis. In recent studies, Wu et al. (2022); Sun T. et al. (2022) provided new insights into the mechanisms involved in CRC progression and metastasis via sEVs. Specifically, Wu et al. found that SW620 cell-derived sEV-miR-224-5p promoted the proliferation, invasion, and malignant transformation of CRC cells by transferring miR-224-5p to CRC cells to target and inhibit CMTM4 expression. Additionally, Sun et al. demonstrated that miR-17-5p is upregulated in CRC and plays a crucial role in promoting CRC development. Specifically, miR-17-5p was delivered to CRC cells via CRC stem cell-derived sEVs and increased PD-L1 expression by inhibiting SPOP, which in turn suppressed anti-tumor immunity and promoted the growth of CRC cells.

Luan et al. (2020) discovered that the lncRNA UCA1 was upregulated in CRC tissues and serum sEVs of CRC patients. UCA1 was shown to upregulate MYO6 expression by sponging miR-143, indicating that UCA1 acts as an oncogene in CRC and that its upregulation promotes CRC cell proliferation and migration. In addition, Li et al. (2022c) found that the lncRNA01315 was overexpressed in CD133/CD44 CRC cells and sEVs and that sEVs overexpressing lncRNA01315 effectively promoted the proliferation, migration, and stemness of colorectal cancer cells. Yin et al. (2021) found that the lncRNA NNT-AS1 was highly expressed in CRC tissues and promoted proliferation, invasion, and migration of CRC cells by sponging miR-496 and regulating downstream RAP2C. These findings highlight the importance of lncRNA-mediated communication in CRC progression and suggest that lncRNAs could serve as potential therapeutic targets and biomarkers for CRC diagnosis and treatment.

**FIGURE 3:**
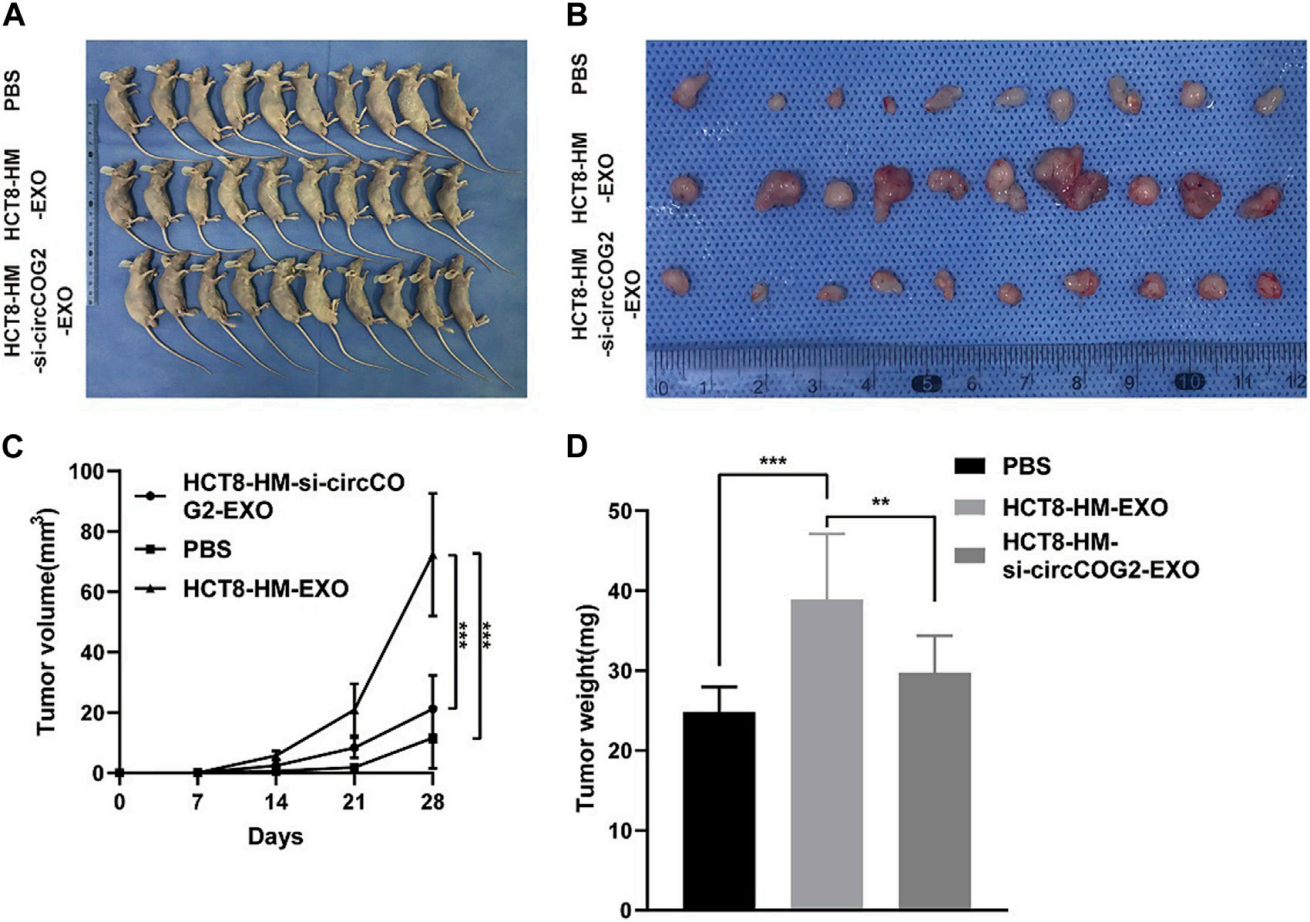
Tumor cell-derived sEV mediates CRC progression *in vivo*. HCT8-HM: Author team establishes CRC cell lines with high metastatic potential; HCT8-HM-EXO: sEV secreted by HCT8-HM cells; HCT8-HM-si-circCOG2-EXO: sEV extracted after knockdown of circCOG2 in HCT8-HM cells. **(A)** From top to bottom group 1, group 2, and group 3 were treated with PBS, HCT8-HM-EX, O, and HCT8-HM-si-circCOG2-EXO respectively in nude mice transplanted tumor models. **(B)** The tumor excised after 4 weeks. **(C)** Tumor volume is measured weekly. **(D)** Tumor weight is measured weekly. After treatment with tumor cell-derived sEV, tumor volume, and weight increased significantly, and this may have been caused by the delivery of circCOG2. Reprinted from Gao L, Tang X, He Q, Sun G, Wang C, Qu H. sEVs-transmitted circCOG2 promotes CRC progression via miR-1305/TGF-β2/SMAD3 pathway. Cell Death Discov. 2021; 7 (1):281. Creative Commons license and disclaimer available from: http://creativecommons.org/licenses/by/4.0/(Gao et al., 2021).

Recent studies have shed light on the critical role of sEV-derived circRNAs in CRC progression and metastasis. Specifically, sEV-circRNAs are associated with signaling pathways involved in tumorigenesis and aggressive features of CRC. It has been found that circRNAs upregulated in CRC act as transforming genes and their activation can cause normal cells to become cancerous, invasive, and metastatic (Vakhshiteh et al., 2021) (Figure 3). Furthermore, CD133 cell-derived sEV-circ-ABCC1 is upregulated and promotes cell stemness and metastasis in CD133/Caco2 or CD133/HCT15 cells. This circRNA can mediate cell stemness and metastasis in CRC by activating the Wnt/β-linked protein pathway to promote CRC progression (Zhao S. et al., 2020). Shang et al. (2020a) identified circ PACRGL, a novel CRC-derived sEV-circRNA that was significantly upregulated in CRC cells. This circRNA was found to affect TGF-β1 expression by sponging miR-142-3p and miR-506-3p, promoting the proliferation, migration, and invasion of colorectal cancer cells while regulating N3-N1 neutrophil differentiation, ultimately leading to CRC progression. Yang et al. (2020) found increased secretion of sEV-circ-133 due to the hypoxic microenvironment, which positively correlated with the disease course and promoted CRC metastasis via GEF-H1/RhoA. Further research is needed to fully elucidate the mechanisms and pathways involved in the transfer of sEV-circ-133 in CRC progression and metastasis. Moreover, Fan et al. (2023) demonstrated that sEV-circ_0000395 is highly expressed in CRC and promotes CRC progression. This circRNA was found to deregulate the malignant phenotype of CRC cells otherwise suppressed by high expression of miR-432-5p by upregulating MYH9. These findings highlight the critical role of sEV-circRNAs and their involvement in CRC pathogenesis and provide a promising avenue for developing novel therapeutic strategies and diagnostic tools.

### 5.2 Induced premetastatic niche formation of pre-metastatic ecotone and promoting CRC liver metastasis

The premalignant niche (PMN) is a complex and dynamic system regulated by intercellular interactions that are responsible for tumor progression and distant metastasis (Zhao X. et al., 2020). sEV-derived RncRNAs are critical cancer cell-derived factors that initiate niche formation before distant organ metastasis (Costa-Silva et al., 2015). Moreover, sEV-RncRNAs have been shown to play an important role in colorectal cancer liver metastasis (CRLM).

sEV-derived miRNAs have been shown to promote tumorigenesis and tumor progression by regulating the interactions between tumor cells and tumor-associated macrophages (TAMs). Zhao H. et al. (2020) demonstrated aberrant high expression of sEV-miR-934 in colorectal cancer liver metastasis (CRLM). Specifically, miR-934 encapsulated in sEVs can contribute to tumorigenesis and tumor progression by secreting CXCL13 to activate CXCL5/CXCR65/NFκB/p934/miR-934 in CRC cells. This, in turn, polarizes M2 macrophages, leading to sustained crosstalk between tumor cells and TAMs, inducing premalignant niche site formation and promoting CRLM. Tian et al. (2021) found that CRC-secreted miR-221 and miR-222 could be transported to hepatic stromal cells via sEVs to induce premalignant niche (PMN) formation and promote CRC cell metastasis. This was achieved by inhibiting serine protease inhibitor, Kunitz type 1 (SPINT1) expression and inducing hepatocyte growth factor (HGF) in PMN. Moreover, Sun J. et al. (2021) found that the hypoxic microenvironment in primary CRC lesions promotes the release of sEVs. Kupffer cells (KCs) secrete sEVs loaded with miR-135a-5p into the liver, which initiates the large tumor suppressor kinase 2-yes-associated protein-matrix metalloproteinase 7 axis to promote the occurrence of CRC liver metastasis.

lncRNA PCAT1 was highly expressed in CRC primary cell lines and their sEVs, and knockdown of PCAT1 resulted in upregulation of Netrin-1 and CD146 expression, downregulation of miR-329-3p expression, increased proliferation and migration capacity of T84 cells, and enhanced F-actin signaling after undergoing co-culture experiments, promoting CRLM (Fang et al., 2022).

sEV-circ PABPC1 was found to be overexpressed in CRC tissues and promote CRLM by acting as an endogenous competing RNA (ceRNA) for miR-874/miR-1292, upregulating ADAM19 and BMP4 expression (Li et al., 2022d).

These findings highlight the critical role of sEV-derived RncRNAs and their involvement in CRC pathogenesis and metastasis, particularly in the context of CRLM. Understanding the underlying mechanisms of sEV-RncRNAs -mediated crosstalk between tumor cells and TAMs could lead to the development of novel therapeutic strategies for CRC. Further research is needed to fully elucidate the mechanisms and pathways involved in the transfer of sEV-RncRNAs and their cargo in CRC progression and metastasis.

### 5.3 Promotion of angiogenesis

sEVs are known to contain various types of RNA molecules that play critical roles in cancer progression and metastasis. Recent studies have shown that sEV-derived RNAs can contribute to angiogenesis, a process that is essential for promoting the growth and survival of tumors, by upregulating the expression of pro-angiogenic factors (Kucharzewska et al., 2013; Xie et al., 2019). The upregulation of pro-angiogenic factors is crucial for promoting angiogenesis, which provides sufficient oxygen and nutrients to support the growth and survival of CRC cells. Therefore, sEV-derived RncRNAs are likely to play an important role in promoting CRC pathogenesis and metastasis by regulating angiogenesis.

CAFs are known to play a critical role in promoting tumor angiogenesis and tumor development in CRC. Many miRNAs are overexpressed in CRC-CAFs and can affect the proliferation, invasiveness, and chemoresistance of neighboring tumor cells through paracrine signaling. Of note, sEV-miR-21-5p is overexpressed in CRC epithelium and adjacent vessels of cancer. This miRNA activates the β-catenin signaling pathway by targeting KRIT1, which in turn promotes angiogenesis and vascular permeability in CRC (He et al., 2021). Zeng et al. (2018) found that sEV-miR-25-3p was upregulated in CRC metastatic sEVs and promoted CRC vascular permeability and angiogenesis by targeting KLF2 and KLF4 to regulate the expression of VEGFR5, ZO-2, occludin, and Claudin4 in endothelial cells. Shang et al. (2020b) revealed that overexpression of miR-183-5p, which targets FOXO1, increased proliferation, invasion, and angiogenesis of CRC cells and may serve as a potential biomarker for CRC.

The adenomatous polyposis coli (APC) gene is known to play a critical role in the pathogenesis of CRC. Recent studies have shown that overexpression of APC can significantly inhibit the proliferation and migration of CRC cells *in vivo* (Wang et al., 2019). Moreover, Wang et al. (2019) demonstrated that silencing of lncRNA-APC1 in APC-overexpressing CRC cells promotes angiogenesis, CRC cell growth, and metastasis by activating the mitogen-activated protein kinase (MAPK) pathway in endothelial cells. These findings suggest that lncRNA-APC1 may play a vital role in modulating the anti-tumor effects of APC in CRC.

Chen C. et al. (2023) showed that sEV-circ TUBGCP4 derived from CRC cells could enhance angiogenesis and promote CRC metastasis by upregulating PDK2 and sponge miR-146b-3p to activate the Akt signaling pathway. This suggests that sEV-circ TUBGCP4 may serve as a potential therapeutic target for CRC. In contrast, circ FNDC3B-rich sEVs were found to inhibit the tumorigenic and angiogenic properties of CRC. Overexpression of miR-937-5p or silencing of TIMP3 reversed this property of circFNDC3B and promoted CRC tumor growth and angiogenesis. These findings suggest that circFNDC3B may also serve as a potential therapeutic target for CRC (Zeng et al., 2020). Further research is needed to fully elucidate the mechanisms involved in the regulation of angiogenesis and CRC metastasis by sEV-circ TUBGCP4 and circ FNDC3B. Understanding these mechanisms could lead to the development of novel therapeutic strategies for CRC.

### 5.4 Promotion of epithelial-mesenchymal transition

Epithelial-to-mesenchymal transition (EMT) is a critical process involved in CRC progression and is associated with the expression of sEV-derived RncRNAs. Recent studies have shown that sEV-RncRNAs can promote the expression of mesenchymal-like cell properties in CRC. Interestingly, EMT-associated sEV-RncRNAs have been identified in CRC and may serve as potential biomarkers for CRC outcomes (Zhang et al., 2021b). These findings highlight the crucial role of sEV-ncRNAs in regulating EMT and CRC progression.

Hypoxia is a crucial factor in promoting EMT in cancer cells. Recent studies have shown that hypoxia decreases the level of sEV-miR-1255b-5p secreted by CRC cells, which in turn upregulates hTERT expression to enhance EMT and telomerase activity, promoting EMT in CRC (Zhang et al., 2020c). Furthermore, overexpression of miR-128-3p has been shown to target FOXO4 inhibition and activate the TGF-β/SMAD and JAK/STAT3 signaling pathways. This can transfer pro-EMT features to CRC cells via the sEV delivery function, promoting CRC progression and EMT (Bai et al., 2021). Metastasis is a significant contributor to CRC mortality and is closely associated with circulating tumor cells (CTCs) undergoing EMT. Recent studies have shown that miR-27b-3p is upregulated in EMT-derived CRC cells and positively correlates with CTC counts. Furthermore, miR-27b-3p has been shown to increase vascular permeability and induce EMT, promoting CTC production and ultimately facilitating CTC-mediated CRC metastasis. This is achieved by attenuating the expression of endothelial calmodulin and p120 (Dou et al., 2021). miR-335-5p was found to be highly expressed in sEVs of high metastatic CRC cell origin and can promote EMT with concomitant Ras signaling by targeting RAS p21 protein activator 1 to induce CRC invasion and metastasis (Sun R. et al., 2021). Zhang W. et al. (2022) found that CAFs-derived sEV-miR-625-3p promotes migration, invasion, EMT, and chemoresistance of CRC cells through the CELF2/WWOX pathway. Mesenchymal stem cell (MSCs)-derived sEVs carrying miR-3940-5p be transferred to CRC cells via intercellular communication. These sEVs upregulate transforming growth factor-β1 signaling by expressing per-ITGA6, promoting CRC cell invasion and EMT (Li C. et al., 2021). These findings demonstrate the critical role of sEV-derived miRNAs in regulating EMT and MET in CRC cells. Moreover, tumor-derived sEVs are effective vectors for the delivery of miR-375 to target cells. This miRNA upregulates the expression of E-calmodulin, downregulates CD44 and CD133, and regulates EMT in CRC cells (Rezaei et al., 2021).

Zhou et al. (2021) found that lncRNA LINC00659 was aberrantly highly expressed in cancer-associated fibroblast (CAF)-derived sEVs. This lncRNA could promote CRC cell proliferation, invasion, migration, and EMT by acting as a sponge for miR-342-3p and upregulating ANXA2 expression.

circ COG2 is highly expressed in CRC tissues, plasma, and sEVs. Upregulation of circ COG2 can target and inhibit miR-1305 expression, promoting EMT of CRC through activation of the TGF-β2/SMAD3 pathway. High expression of circ COG2 is associated with poor prognosis, highlighting its potential as a therapeutic target for colorectal cancer (Gao et al., 2021). Furthermore, the knockdown of circ COG2 has been shown to inhibit CRC cell viability, further emphasizing its potential as a therapeutic target for the treatment of CRC.

### 5.5 Induction of immune escape

Tumor-derived sEVs can play an important role in immune escape and immune cell differentiation in colorectal cancer by participating in mechanisms of immune cell targeting and interaction through immune activation, immune surveillance, and regulation of antigen presentation in immune regulation of cancer cells (Xie et al., 2019).

**FIGURE 4:**
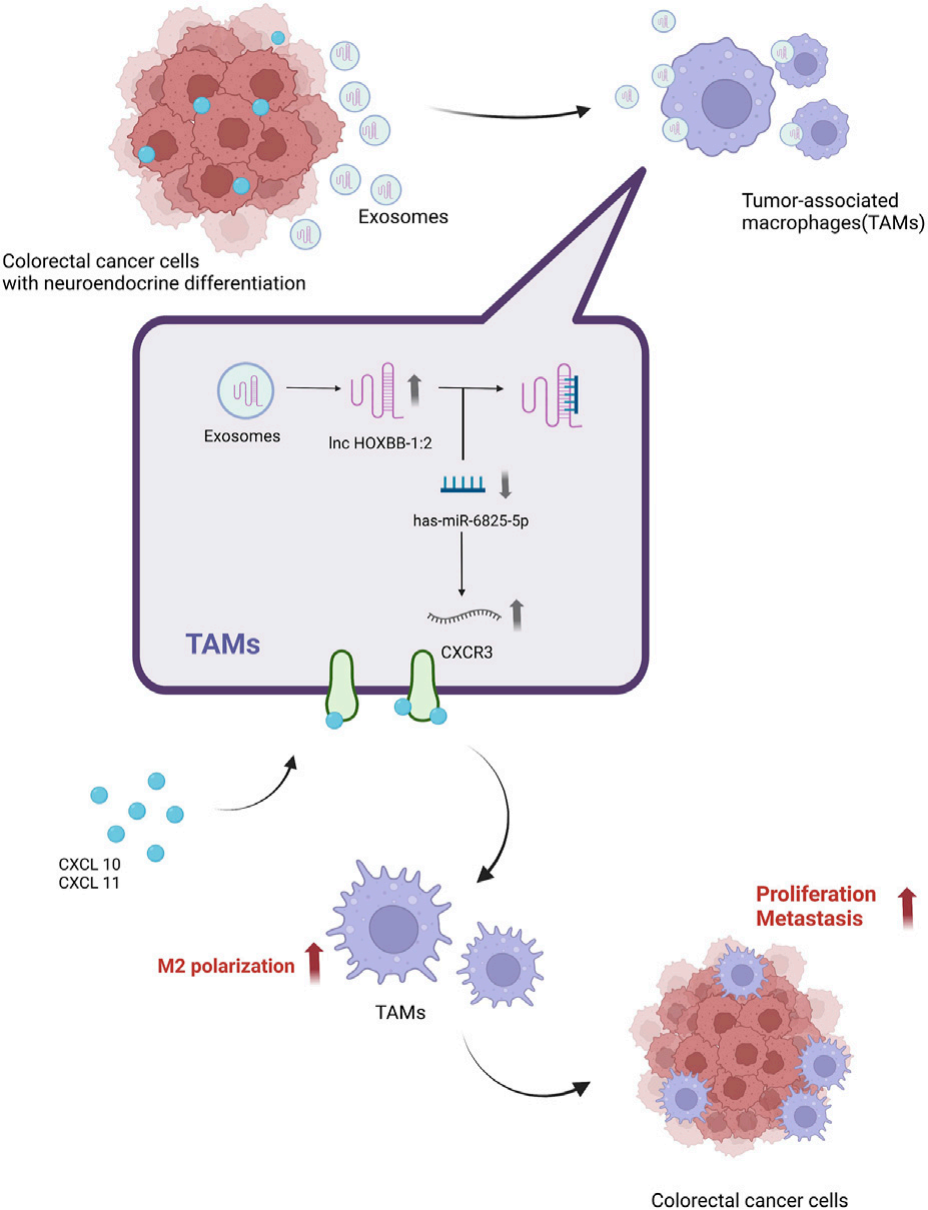
A possible mechanism for lnc-HOXB8-1:2 to promote colorectal cancer progression via lnc-HOXB8-1:2/has-miR-6825-5p/CXCR3 axis. Neuroendocrine differentiation (NED) is a risk factor for CRC progression and death. Neuroendocrine differentiated colon cancer cell-derived sEV-lnc-HOXB8-1:2 was taken up by Tumor-associated macrophages (TAMs) *in vitro*. Uptake by TAMs increases expression in TAMs and promotes TAMs infiltration and M2 polarization by positively regulating CXCR3 expression as a ceRNA for has-miR-6825-5p, which in turn leads to neuroendocrine differentiated CRC progression (Li X. et al., 2022). Created by BioRender.com.

Huang et al. (2021) found that sEV-lncRNA SNHG10 expression was upregulated in CRC, and upregulation of INHBC expression by SNHG10 significantly inhibited the viability and cytotoxicity of natural killer cells (NK cells), causing CRC cells to immune escape from NK cells and promoting the growth of colorectal cancer. sEV-lncRNA suppresses CD8 T cell activity and promotes differentiation of Th17 cells, Treg and Bregs to regulate the adaptive immune response in cancer (Zhang et al., 2022b). Sun X. et al. (2021) found that The T helper 17 cells (Th17) in the tumor microenvironment play an important role in the development of CRC. sEV- lncRNA CRNDE-h inhibits the binding of RORγt to E3 ubiquitin ligase Itch by binding to the PPXY motif of RORγt, thus hindering the ubiquitination and degradation of RORγt and ultimately promoting the differentiation of Th17 cells. It was found that LncRNA KCNQ1OT1 expression was significantly increased in CRC tissues and promoted tumor development through co-sEVs subsecretion, and overexpression of KCNQ1OT1 significantly inhibited T cell-mediated cell killing induced immune escape, inhibited USP22 expression through competitive binding of miR-30a-5p, regulated PD-L22 ubiquitination, and thus promoted immune escape in colorectal cancer (Xian et al., 2021). The sEV-lncRNA HOTAIR secreted by tumor cells can be delivered to the surrounding B cells, leading to the expression of PDL1 by B cells, thus suppressing the immune response of T cells and promoting the growth and metastasis of colorectal cancer. Inhibiting the secretion of sEVs or targeting HOTAIR/PDL1 can reverse the immunosuppressive effect of B cells, thus enhancing the immune response of T cells (Xie et al., 2023). The lncRNA RPPH1 was found to be significantly upregulated in CRC tissues and its expression was upregulated by binding to β-III tubulin (TUBB3), which promotes EMT in CRC cells, and also transports RPPH1 into macrophages via sEVs, mediating macrophage M2 polarization and thus promoting CRC cell metastasis and proliferation (Liang et al., 2019). The presence and oncogenic role of sEV-lncRNA in neuroendocrine differentiation (NED) (Figure 4).

### 5.6 Induction of drug or radioresistance

Drug resistance triggers relapse and shortens patient survival. The coordinated role of tumor-secreted sEVs is critical for cell crosstalk and pharmacological resistance promotion. sEV-RncRNAs can induce drug resistance in colorectal cancer cells through different mechanisms (De Los Santos et al., 2019).

Chen et al. (2021) found that cancer-associated fibroblasts (CAFs) derived miR-590-3p could be transferred to CRC cells via sEVs, reducing CLCA4 expression and enhancing radioresistance in CRC. This highlights the critical role of sEV-derived miRNAs in regulating the response to radiation therapy in CRC. In addition, Hu et al. (2019) found that CAFs can transfer sEVs into CRC cells, leading to a significant increase in intracellular miR-92a-3p levels and ultimately promoting CRC cell stemness, colorectal cancer metastasis, and chemoresistance. Chemoresistance is a major challenge in treating CRC, and targeting regulatory T cells (Tregs) is an effective way to improve chemosensitivity. Circulating miR-208b has been shown to inhibit programmed cell death factor 4 (PDCD4) expression, leading to Treg differentiation that induces CRC tumor growth and oxaliplatin resistance (Ning et al., 2021). Chen B. et al. (2020) found that miR-93-5p, a novel sEV cargo, was upregulated and delivered to CRC cells via sEVs to target and inhibit FOXALIPLATIN1, thereby inhibiting CRC cell apoptosis and induce radioresistance in CRC cells. Moreover, abundant miR-24-3p in CAFs-derived sEVs was found to upregulate HEPH expression by targeting CDX2, inducing methotrexate resistance in CRC cells (Zhang et al., 2021c). In addition, sEV-miR-181d-5p derived from cancer-associated fibroblasts was found to be associated with 5-fluorouracil (5-FU) resistance. NCALD was found to be negatively correlated with 5-FU sensitivity, and miR-181d-5p overexpression reduced the chemosensitivity of CRC cells to 5-FU by downregulating NCALD expression (Pan S. et al., 2022). Another study found that miR-21, which is highly expressed in CRC sEVs, increased the resistance of CRC cells to 5-FU by downregulating the target gene PDCD4 (Sun et al., 2020).

Han et al. (2017) found that sEV-lncRNA CRNDE was overexpressed in CRC tissues and that knockdown of CRNDE resulted in suppression of target miR-181a-5p overexpression in CRC cells, which in turn inhibited CRC cell proliferation and reduced chemoresistance. Chen H. L. et al. (2020) found that sEV-lncRNA HOTTIP was associated with mitomycin resistance in CRC cells. lncRNA HOTTIP was upregulated in mitomycin-resistant CRC cells and promoted mitomycin resistance in CRC cells by upregulating KPNA3 expression through sponge miR-214. Yuan et al. (2022) found that LncRNA UCA1 could induce cetuximab resistance in CRC cells by sponging miR-495 and upregulating the expression of HGF and c-MET, which were originally inhabited by miR-495. Deng et al. (2020) showed that lncRNA CCAL can be used to transfer sEVs from CAFs to CRC cells and increase β-linked protein mRNA and protein levels by interacting with the mRNA stabilizing protein HuR, activating the β-linked protein pathway and thus promoting Oxaliplatin resistance in CRC cells.

CRCs usually rely on aerobic glycolysis to produce ATP for rapid growth and chemoresistance, sEVs derived from Oxaliplatin-resistant cells deliver ciRS-122 to sensitive cells and promote PKM2 expression at the post-transcriptional level by sponging miR-122, thereby accelerating glycolysis and reducing drug sensitivity in sensitive CRC cells (Wang X. et al., 2020). circ ATG4B was found to be associated with CRC Oxaliplatin resistance, circ ATG4B expression was upregulated in Oxaliplatin-resistant CRC cells and caused dissociation of ATG4B from TMED10 by acting as a decoy for TMED10, which led to increased autophagy and increased Oxaliplatin resistance in CRC (Pan Z. et al., 2022). Wang Y. et al. (2020) found that pyruvate kinase M2 isoform (PKM2) was overexpressed in CRC Oxaliplatin-resistant cells and that sEVs-derived ciRS-122 could act as a sponge for miR-122 to influence the expression pattern of PKM2 and positively regulate PKM2 resistance, altering the resistance of colorectal cancer cells to Oxaliplatin by inhibiting the ciRS-122/miR-122/PKM2 axis. CAFs-exo-derived circ N4BP2L2 promotes CRC cell stemness and oxaliplatin resistance by upregulating EIF4A3 expression and thereby regulating the PI3K/AKT/mTOR axis (Qu M. et al., 2022). sEV-circ_0000338 has high levels and plays an important role in the serum of 5-FU resistant CRC patients, circ_0000338 also enhances 3-FU resistance in CRC by sponging miR-5 and miR-217-485p, providing a better diagnostic and therapeutic intervention for 5-FU resistance in CRC patients (Zhao et al., 2021). circ_IFT80 is overexpressed in CRC cells and has a tumor-promoting and radiosensitizing effect, and reverses the tumor-promoting effect of circ_IFT80 by inhibiting MSI1 expression through the sponge miR-296-5p, which inhibits CRC development and promotes the promotion of radiosensitivity in CRC cells (Li L. et al., 2021). sEV-circCOG2 is highly expressed in CRC tissues, plasma, and sEVs and is associated with poor prognosis. Upregulation of circCOG2 can target miR-1305 expression and promote EMT for CRC by activating the TGF-β2/SMAD3 pathway, and knockdown of circCOG2 can inhibit the viability of CRC cells, which can be a potential therapeutic target for colorectal cancer (Gao et al., 2021).

### 5.7 The role of infected bacterial sEVs in CRC progression

Mounting evidence suggests that bacteria play a crucial role in the pathogenesis of CRC. Dysbiosis in the gut microbiome can lead to chronic inflammation and the production of oncogenic metabolites, ultimately resulting in tumor development (Jun, 2022). Parvimonas micra (P. micra) has been shown to suppress PTPRR expression, consequently activating the Ras/ERK/c-Fos signaling pathway and promoting CRC cell proliferation by upregulating miR-218-5p transcription in cancer patients’ feces (Chang et al., 2023). Guo et al. (2020) found that *Fusobacterium* nucleatum (Fn) infection may induce the production of abundant miR-1246/92b-3p/27a-3p and CXCL16/RhoA/IL-8-containing sEVs in tumor cells, which can be transferred to non-infected cells via these sEVs, promoting CRC metastasis.

Lu et al. (2022) have demonstrated that Fn can promote CRC metastasis by upregulating long non-coding RNA (lncRNA) EVADR expression, making EVADR a modular scaffold for Y-box binding protein 1 (YBX1), which in turn directly enhances the translation of epithelial-mesenchymal transition (EMT)-related factors. These findings illustrate the critical role of Fn in the regulation of lncRNAs and EMT-related factors, providing new insights into the molecular mechanisms underlying CRC metastasis. Cao et al. (2021) found that sEV-miR-149-3p derived from Enterotoxigenic *Bacteroides fragilis* (ETBF) treated cells promoted T helper type 17 cell differentiation and promoted CRC cell proliferation by downregulating miR-149-3p.

## 6 Progress of sEV-RncRNAs for CRC

CRC is the leading cause of cancer deaths worldwide, and there is an increasing focus on the role of sEV-RncRNAs in the treatment of CRC, identifying patients with poor prognosis or high risk of recurrence, which may allow us to selectively intensify therapy (Yamamoto et al., 2021). The standard treatment for CRC is radical resection combined with adjuvant chemotherapy (Du et al., 2022), chemotherapy and radiotherapy is the standard of care for locally advanced disease, targeting Indoleamine 2,3 dioxygenase 1 (IDO1) can improve the effect of radiotherapy and slow down the growth of CRC tumors (Chen et al., 2020c). Qian et al. (2022) successfully designed sEVs nanoprobes for combined antitumor chemotherapy and photodynamic therapy using tumor-derived sEVs loaded with doxorubicin (Dox) and the photodynamic therapeutic agent 5-aminolevulinic acid, enabling the visualization of targeted transport of multiple drugs and tumor sEVs. However, for some patients, chemotherapy may promote tumor metastasis by inducing the secretion of EVs. Wills et al. (2021) demonstrated that EVs secretion is a mechanism by which chemotherapy promotes metastasis, and low doses of Dox promote breast cancer metastasis by upregulating EVs secretion and inducing PHN formation. Shen et al. (2019) found that chemotherapy-induced breast cancer cells secrete multiple EV-miRNAs that reprogrammed BC cells by targeting the transcription factor One Cut Homeobox 2 (ONECUT2), which in turn induced cancer stem cell-like cell (CSC) phenotype, and that cutting off this pathway could improve the anticancer effect of breast cancer chemotherapy. Keklikoglou et al. (2019) found that paclitaxel and anthracyclines induced breast cancer to secrete pro-metastatic EVs through annexin-A6 (ANXA6), which induced the formation of PHN and thus promoted lung metastasis of breast cancer. The above evidence suggests that chemotherapy induces the secretion of EVs and modulates the tumor phenotype. It was found that tumor sEVs-based nanoparticles are effective drug carriers for chemotherapy, and to improve the efficacy of chemotherapy, Tu et al. successfully developed biocompatible exosomal Porous silicon nanoparticles (PSiNPs) for targeted cancer chemotherapy, which, when loaded with chemotherapeutic drugs, can achieve targeted elimination of tumor cells and have the potential to improve anticancer efficacy (Yong et al., 2019). sEVs play an important role in the treatment (Table 4) and prognosis (Table 5) of CRC, therefore, choosing the right combination of drugs for each patient’s intestinal environment and the right treatment strategy is the key to successful treatment (Wozniakova et al., 2022). Milk-derived extracellular vesicles can play an important role in intercellular information transfer as high-quality nanocarriers (Babaker et al., 2022), encapsulation of anthocyanins in Milk-derived extracellular vesicles enhances the anti-proliferative and anti-inflammatory effects on various cancer cells without toxic side effects, achieving better efficacy (Munagala et al., 2017). Cui et al. (2020) found that miR-1539 was overexpressed in CRC tissues and serum of CRC patients, suggesting changes in tumor location and poor prognosis, and could be used as a novel potential biomarker for CRC screening.

**TABLE 4:** Early evidence of therapeutic sEVs for CRC treatment.

EVs	Cargoes	Targets	Treatment	References
sEV-miRNAs	miR-375-3p	Thymidylate synthase (TYMS)	Possible new therapeutic strategy for enhanced chemosensitivity to 5-FU in the future	Xu et al. (2020b)
	miR-203	TYMS	Enhanced 5-FU chemosensitivity	Li et al. (2015)
	miR-154-5p	lncRNA SNHG1	As a potential target for the diagnosis and treatment of colorectal cancer	Xu et al. (2018)
	miR-21-5p	PTEN	Promotes immune escape of tumors	Yin et al. (2022)
	miR-200a			
sEV-lncRNAs	lncRNA CRNDE	miR-181a-5p	Regulation of CRC progression and chemoresistance	Han et al. (2017)
sEV-circRNAs	circ_0007334	miR-12	Impedes colorectal cancer tumor growth and angiogenesis *in vivo*	Bai et al. (2022)
	circ_0094343	miR-766-5p	Improved its chemosensitivity to various chemotherapeutic agents	Li et al. (2022a)
	circEPB41L2	miR-21–5	Inhibit the growth of colorectal cancer tumors	Jiang et al. (2021a)
		miR-942-5p		
	circ_0000338	miR-217	Enhanced 3-FU resistance in CRC	Zhao et al. (2021)
		miR-485-3p		

**TABLE 5:** Key evidence for the prognostic role of sEVs in CRC.

sEVs	Cargoes	Expression	Type of biomarker	Role	References
sEV-miRNA	miR-19a	Upregulated	Prognostic	The reduced survival rate in CRC patients suggests a poor prognosis	Matsumura et al. (2015)
	miR-1539	Upregulated	Prognostic	Suggests changes in tumor location and poor prognosis	Cui et al. (2020)
	miR-25-3p	Upregulated	Prognostic	Promotes angiogenesis	Zeng et al. (2018)
	miR-152-3p	Upregulated	Prognostic	For drug development and as a prognostic biomarker	Lai et al. (2021)
	miR-934	Upregulated	Prognostic	Promotion of CRC liver metastasis	Zhao et al. (2020b)
sEV-lncRNA	lncRNA RPPH1	Upregulated	Prognostic	Promotes CRC cell migration, invasion, and EMT	Liang et al. (2020b)
sEV-circRNA	circCOG2	Upregulated	Prognostic	Promotes CRC proliferation, migration, and invasion	Gao et al. (2021)

### 6.1 Inhibit cancer cell proliferation and promote apoptosis of cancer cells

Numerous studies have demonstrated that sEV-RncRNAs are involved in the regulation of the CRC anti-apoptotic signaling pathway in the tumor microenvironment, thereby inhibiting the proliferation of CRC cells and hindering their development (Nabariya et al., 2020).

Ji et al. (2021) found that miRNA-129-5p could be delivered to target cells via sEVs to inhibit CRC tumor invasion and migration and promote CRC cell apoptosis. Han et al. (2021) found that sEVs loaded with anti-miRNA-221 oligonucleotides were taken up by colon cancer cells through NRP-1 protein and downregulated caco2 and HCT116 in CRC cells, which could inhibit CRC cell proliferation. In addition, sEVs isolated from starved CT-26 cells delivered miR-34a to tumor cells and were able to induce apoptosis and inhibit the migration of CRC cells (Hosseini et al., 2021). It was also found that overexpression of miR-193a, which inhibits CRC cell proliferation, along with downregulation of let-7g, can improve survival rates (Cho et al., 2021). The pathogenesis and prevention of CRC are closely related to dietary composition. Milk sEVs are rich in miR-27b, miR-15b and miR-148a. miR-27b has higher cytotoxic effects, increases mitochondrial ROS content and lysosomal accumulation, and promotes apoptosis of CRC cells (Martino et al., 2022). Chen et al. (2020d) found that bone marrow mesenchymal stem cell-derived sEV-miR-4461 attenuated the proliferation and migration of HCT116 and SW480 cells by suppressing COPB2 expression, which in turn inhibited the development of CRC. In addition, Xuan and Wang. (2023) found that sEV-miR-506-3p was lowly expressed in CRC tissues and downregulated GSTP1 expression by transferring miR-506-3p-transfected FHC cells to SW480 cells, which in turn induced apoptosis in CRC cells. Qu Z. et al. (2022) found that miR-431-5p was downregulated in CRC and negatively correlated with PRDX1 expression and that human umbilical cord mesenchymal stem cells (hUCMSCs)-derived sEVs negatively regulated PRDX1 expression by upregulating miR-431-5p, which in turn inhibited CRC cell growth. Chen J. et al. (2023) also demonstrated that hUCMSCs-derived sEVs regulate SUCNR1 expression through mediating miR-1827 and inhibit macrophage polarization, which in turn inhibits CRC cell proliferation and liver metastasis; Liu J. et al. (2021) found that miR-140-3p was downregulated and acted as a tumor suppressor in CRC tissues and cellular sEVs, which was achieved by miR-140-3p overexpression suppressing the expression of BCL9 and BCL2 in HCT116 cells. miR-198 downregulated the expression of fucosyltransferase 8 (FUT8) and suppressed the proliferation and invasion of colorectal cancer (Wang et al., 2014). Yan et al. (2020) found that sEV-miR-548c-5p could inhibit CRC cell invasion and growth, which was achieved by targeting HIF1A to regulate the expression of CDC42 in CRC cells. Wang and Lin. (2021) demonstrated that sEV-miR-22-3p was downregulated in CRC tissues and cells, inhibiting the PI3K/AKT pathway through negative regulation of RAP2B, which in turn inhibited CRC cell proliferation and invasion. Jahangiri et al. (2022) found that MSCs-derived sEVs were enriched in miR-100 and miR-143 and could induce apoptosis in CRC cells via the miR-100/mTOR/miR-143 axis. Di et al. (2021) found that CRC cell-derived sEVs were able to increase white adiposome browning and thus induce fat loss in CRC cachexia by releasing abundant miR-146-5p, which inhibits the downstream gene containing the cis-domain C10.

Lai et al. (2021) found that sEV-lncRNA Plasmacytoma variant translocation 1 (PVT1) was associated with colon cancer incidence, disease recurrence, and distant metastasis and was regulated by miR-152-3p. increased miR-152-3p expression reduced tumorigenesis and the regulatory pathway of miR-152-3p targeting PVT1 could be used for drug development and as a prognostic biomarker. Han et al. (2017) found that lncRNA CRNDE was upregulated in CRC tissues and was able to inhibit CRC cell proliferation and improve chemosensitivity by upregulating miR-181a-5p expression.

Zheng et al. (2022) found that circLPAR1 correlated with overall survival in colorectal cancer patients. sEV-circLPAR1 was internalized by CRC cells and directly bound to eIF1h specifically inhibiting METTL3-eIF3h translation and suppressing the expression of oncogene BRD4, which in turn inhibited the growth of CRC cells. In addition, the knockdown of CAFs-derived sEV-circN4BP2L2 similarly inhibited CRC cell genesis and metastasis (Yang Y. et al., 2022). Yang et al. (2021) found that CAFs can release circEIF3K under hypoxia, and circEIF3K is upregulated in late CRC. circEIF3K knockdown increased miR-214 levels in CRC cells, and miR-214 attenuated proliferation, invasion, and metastasis of CRC cells under hypoxic conditions. Chen et al. (2022) found that circRHOBTB3 has an inhibitory effect on tumor cell proliferation by targeting the negative cyclization element of circRHOBTB3 through antisense oligonucleotides (ASOs), to increase the expression of circRHOBTB3 and thus inhibit CRC progression. Jiang et al. (2021a) found that circEPB41L2 was downregulated in sEVs of colorectal cancer patients and cells, and overexpression of circEPB41L2 could inhibit the activity of PTEN/AKT signaling pathway, miR-21-5p or miR-942-5p could deregulate the activity of TEN/AKT signaling pathway inhibited by circEPB41L2, and by downregulating miR-21-5p or miR-942-5p could promote CRC cell apoptosis and inhibit CRC progression. Another study by this author also confirmed the upregulation of circ-RNF121 expression in CRC, knockdown of circ-RNF121 inhibited cell proliferation and promoted apoptosis, and downregulation of FOXM1224 expression by sponging miR-1224-5p was able to inhibit CRC cell growth (Jiang et al., 2021b). Bai et al. (2022) found that circ_0007334 expression was upregulated in CRC cell-derived sEVs and could play a role in inhibiting CRC cell viability and angiogenesis by suppressing miR-12 to regulate KLF577 expression.

### 6.2 Increased chemotherapy and radiation sensitivity

Therapeutic resistance has been a major obstacle to the successful treatment of CRC. sEVs, as natural carriers, enable targeted drug transport and enhance the therapeutic effect by modulating ncRNAs in CRC and improving the chemosensitivity and radiosensitivity of drug-resistant cells (Lampropoulou et al., 2022).

**FIGURE 5:**
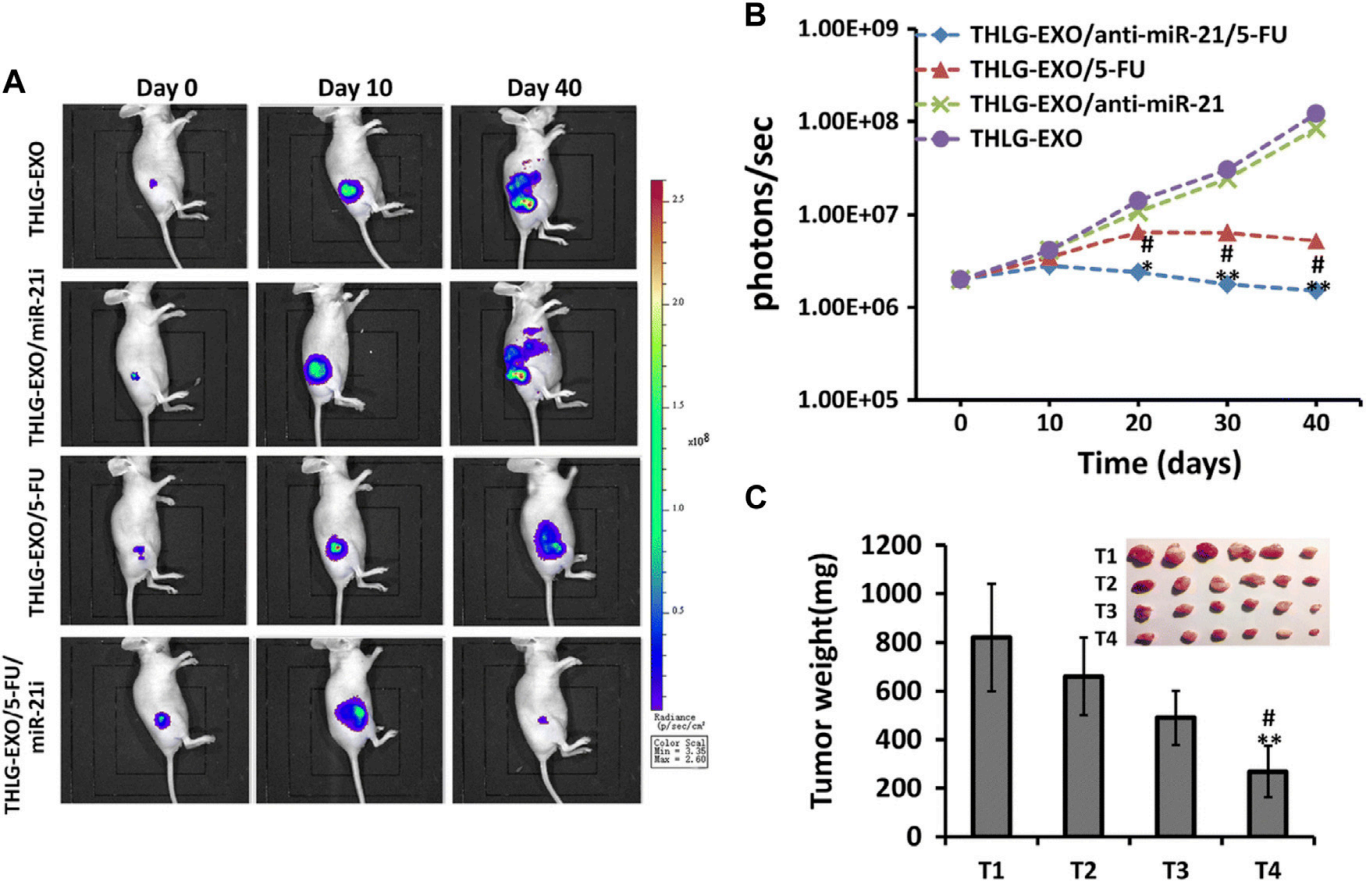
Anti-colorectal tumor effects of engineered sEV *in vivo*. HLG-EXO: The author team produced a kind of engineering sEV; THLG-EXO/miR-21i: Engineered sEV encapsulated with miR-21 inhibitor; THLG-EXO/5-FU: Engineering sEV for encapsulating chemotherapeutic drug 5-FU; THLG-EXO/5-FU/MIR-21i: Simultaneous packaging of 5-FU with miR-21i for engineering sEV. **(A)** THLG-EXO, THLG-EXO/miR-21i, THLG-EXO/5-FU, and THLG-EXO/5-FU/MIR-21i were used to treat 5-FU resistant tumorigenic models in nude mice. Bioluminescence images of tumor growth at different time points. **(B)** Mean bioluminescence intensity of tumor sites in each group. **(C)** Tumor weights for each group. T1-T4 represent THLG-EXO, THLG-EXO/miR-21i, THLG-EXO/5-FU, and THLG-EXO/5-FU/miR-21i groups respectively. Reprinted from Liang G, Zhu Y, Ali DJ, et al. Engineered sEVs for targeted co-delivery of miR-21 inhibitor and chemotherapeutics to reverse drug resistance in colon cancer. J Nanobiotechnology. 2020; 18 (1):10. Creative Commons license and disclaimer available from: http://creativecommons.org/licenses/by/4.0/(Liang G. et al., 2020).

Sun W. et al. (2022) found that CRC-derived sEVs could act as vectors to deliver miR-19b to CRC cells, inhibit miR-19b radioresistance to CRC by upregulating FBXW7 expression, and improve sensitivity to radiotherapy. Yao et al. (2020) found that HEK293T cell line stably expressing miR-204-5p could transfer sEV-miR-204-5p to CRC cells via paracrine action and inhibit CRC progression and enhance CRC chemosensitivity by targeting Bcl2 and RAB22A. Drug resistance to 5-FU is one of the main reasons for failure in colorectal cancer. Delivery of 5-FU and miR-21i packaged as engineered sEVs to cancer cells silences miR-21i expression, inhibits cell proliferation, induces apoptosis, and can upregulate chemosensitivity to 5-FU, improving the therapeutic efficiency of CRC (Figure 5) (Liang G. et al., 2020). Transcriptional inhibitor GFI1B induces low expression of PGM5 antisense RNA 1 (PGM5-AS1) in colon cancer and inhibits CRC growth and oxaliplatin resistance, packaging oxaliplatin and PGM5-AS1 into engineered sEVs, reversing drug resistance in recipient cells, and increasing chemosensitivity of CRC cells to oxaliplatin (Hui et al., 2022). Xiao et al. (2021) found that sEV-miR-1915-3p was overexpressed in non-tumorigenic intestinal cell lines and delivered via sEVs to oxaliplatin-resistant CRC cells by inhibiting the EMT-promoting oncogenes 6-phosphofructo-2-kinase/fructose-2,6-bisphosphatase 3 (6-phosphofructo-2 kinase/fructose-2,6-biphosphatase 3 (PFKFB3) and ubiquitin carboxyl-terminal hydrolase 2 (USP2) decreased the expression of oxaliplatin-resistant CRC cell lines and *in vivo* EMT markers by inhibiting expression and increased the chemosensitivity of CRC cells to oxaliplatin.

Li C and X. (2022) found that circ_0094343 was downregulated in CRC tissues and targeted TRIM67 expression by sponging miR-766-5p to inhibit glycolysis and improve chemosensitivity of CRC cells to 5-FU, oxaliplatin, and DOX. Wang et al. (2021) found that sEV-circ-0067835 was upregulated in the serum of CRC patients after radiotherapy, and regulated IGF1R expression through the sponge miR-296-5p, which in turn inhibited CRC growth and enhanced CRC cell apoptosis and radiosensitivity; Yang M. et al. (2022) found that circ PTPRA downregulates and inhibits CRC cell proliferation in CRC, and increases SMAD4 expression through circ PTPRA overexpression via sponge miR-671-5p, which inhibits malignant growth and promotes radiosensitivity of CRC cells; Xu H. et al. (2021) found that circ-FBXW7-transfected FHC cells secreted by circ-FBXW7 transferred to drug-resistant CRC cells via sEVs improved chemoresistance to oxaliplatin in CRC by directly binding miR-128-3p. Zhang et al. (2022c) found that circ_0006174 was highly expressed in DOX-resistant CRC tissues and cells and could enhance CRC sensitivity to DOX by targeting the miR-2/CCND0006174 axis with circ_1205-enriched sEVs using intercellular signaling of sEVs. Wang et al. (2023) found that sEV-circ-0004585 was overexpressed in 5-FU-resistant CRC tissues and cells, and knockdown of circ-0004585 increased 5-FU sensitivity.

## 7 Conclusion and prospects

In recent years, the functions and roles of sEVs in CRC have become clearer, enriching research progress in this field. sEVs, with their wide source, rich content, stable structure, and diverse functions, act as important material carriers that transmit signals between cells and participate in immune regulation of tumors and cancer cells. Different sources of sEVs play distinct roles and exhibit a dual regulatory effect on cancer cells. Of note, non-coding RNAs in sEVs, including miRNA, lncRNA, and circRNA, have crucial roles in CRC development and can serve as potential biomarkers for early detection and diagnosis. Based on extensive studies, sEV-derived ncRNAs have a dual regulatory effect on CRC growth and drug sensitivity, and play significant roles in promoting angiogenesis, immune escape, and EMT. The in-depth study of sEVs-RncRNAs provides a more sensitive and specific approach for early detection and treatment of CRC, enabling targeted transport functions to achieve more accurate drug therapy and timely response to poor prognosis. Furthermore, it offers a greater variety of diagnostic and therapeutic options for CRC. Looking forward, chemotherapy-elicited EVs may provide biomarkers for predicting metastasis risk associated with neoadjuvant chemotherapy in patients who do not achieve a complete response. Thus, continued research on sEVs and their ncRNAs could lead to the development of novel therapeutic strategies and personalized treatments for CRC.

Despite substantial progress in the study of sEVs-RncRNAs in CRC, many questions remain unanswered. For instance, chemotherapy treatment is currently the primary approach for CRC treatment, but traditional chemotherapy drugs can lead to drug resistance and tumor recurrence after chemotherapy due to their induced secretion of sEVs. To address this issue, researchers can further develop engineered sEVs or leverage nanotechnology to transform sEVs into efficient subtargeted drug carriers, thereby enhancing therapeutic efficacy. Moreover, compared with sEVs-RNAs, the genomic DNA of sEVs has been studied relatively little. The intervention and regulation of sEVs on diseases require deeper exploration, and many secrets about sEVs have yet to be uncovered. Thus, continued research is necessary to better understand the role of sEVs in CRC and to develop effective therapeutic strategies. The cost problem and the separation and purification of sEVs also require further investigation and resolution. Gradual breakthroughs in these areas could enable a qualitative leap in sEVs research, facilitating their use as diagnostic and therapeutic tools for various diseases. Overall, although sEVs research is in its infancy, their potential as diagnostic and therapeutic agents has stimulated research in this field. Continued investigation and innovative approaches will be crucial for unlocking the full potential of sEVs and advancing our understanding of their role in CRC and other diseases.
